# 

**Published:** 1965-01

**Authors:** 


### Contents

ATAXIA AND HYPOTHYROIDISM. . . *B. Ashworth* 1

CHROMOSOME ABNORMALITY AS A POSSIBLE CAUSE OF HABITUAL ABORTION . . . . *L. Wingate* 5

TRANSLOCATION MONGOLISM ARISING DE NOVO?. . . . . . . . . *P. W. Short* 7

ARHINENCEPHALY — A SHORT REVIEW *T. F. Draisey, et al.* 13

APPOINTMENTS . . . . . . . . . 17

